# Molecular Mechanisms of ZnO Nanoparticle Dispersion in Solution: Modeling of Surfactant Association, Electrostatic Shielding and Counter Ion Dynamics

**DOI:** 10.1371/journal.pone.0125872

**Published:** 2015-05-11

**Authors:** Patrick Duchstein, Theodor Milek, Dirk Zahn

**Affiliations:** Lehrstuhl für Theoretische Chemie/Computer-Chemie-Centrum Friedrich-Alexander-Universität Erlangen-Nürnberg, Nägelsbach Str. 25, Erlangen, Germany; University of Calgary, CANADA

## Abstract

Molecular models of 5 nm sized ZnO/Zn(OH)2 core-shell nanoparticles in ethanolic solution were derived as scale-up models (based on an earlier model created from ion-by-ion aggregation and self-organization) and subjected to mechanistic analyses of surface stabilization by block-copolymers. The latter comprise a poly-methacrylate chain accounting for strong surfactant association to the nanoparticle by hydrogen bonding and salt-bridges. While dangling poly-ethylene oxide chains provide only a limited degree of sterical hindering to nanoparticle agglomeration, the key mechanism of surface stabilization is electrostatic shielding arising from the acrylates and a halo of Na^+^ counter ions associated to the nanoparticle. Molecular dynamics simulations reveal different solvent shells and distance-dependent mobility of ions and solvent molecules. From this, we provide a molecular rationale of effective particle size, net charge and polarizability of the nanoparticles in solution.

## Introduction

Nanoparticle stabilization and functionalization constitute key steps to nanomaterial production. To account for the hindering of nanoparticle agglomeration, the role of stabilizing molecules has been characterized by two intuitive concepts: the uptake of solutes may be avoided by i) sterical hinderance and ii) electrostatic shielding. The same mechanistic concepts are commonly discussed to account for stabilizing dispersions of nanoparticles in solution. Both mechanistic pictures are well-established from experiments, e.g. by comparing particle size and hydrodynamic radius and by assessing the net charge of colloids from zeta-potentials [1,2].

In-depth understanding of the underlying processes on the molecular scale, that is the mechanisms of surfactant association to the particle, the structuring of solvent shells and the distribution of counter ions near a nanoparticle, is rather sparse. While hard to access experimentally, molecular simulations in principle appear very suited to tackle this issue. However, even when employing computationally efficient molecular models (rather than quantum chemistry approaches) the limited time- and length scales still inherent to molecular simulations call for smart simulation techniques. Such an approach was recently presented by Vlugt and coworkers using the example of metal nanoparticles stabilzed by alkylthiols [3,4]. While this provided profound insights into nanoparticle dispersion on the basis of purely sterical hindering, in the present work we report on molecular simulations related to nanoparticle stabilization from the interplay of steric and electrostatic shielding. For this purpose, we focus on zinc oxide nanoparticles in ethanolic solution stabilized by partially charged block-copolymers, thus referring to a widely used nanosystem [5,6].

## Models and Methods

Molecular simulations related to ZnO nanoparticles synthesized from the vapor may be started from simply cutting hexagonal rods off the single crystal structure [7]. However, ZnO nucleation from solution typically leads to ZnO/Zn(OH)_2_ core/shell nanoparticles with more complex structures. To provide realistic models we build on a recent simulation study related to zinc and hydroxide ion association in ethanolic solution [8], ripening reactions and the nucleation of aggregates comprising ~100 ions. From this, a scale-up model of a ZnO/Zn(OH)_2_ core/shell nanoparticle was prepared as described in detail as supplementary information (S1 Text). The surfactant molecules were chosen as small polymers, namely poly(ethylene oxide-block-methacrylate), (EO_8_-b-MAA_8_)^8-^. While the polyethylenoxide chain (PEO) is uncharged, each of the acrylate units exhibits a -1 charge, thus resulting in a net charge of -8 per surfactant molecule. The MAA_8_ block is chosen for its good binding affinity to ZnO, whilst the EO_8_ block shows poor affinity to the nanoparticle (and is thus expected to be dangling into the solution).

Molecular dynamics simulations were performed on the basis of empirical interaction potentials adopted from earlier studies of ZnO nucleation from solution [8] and bismuth oxide association to polyacrylate [9] (cf. supplementary information: S1 Text, S1 Table and S2 Table, S3 Fig). The simulation temperature and pressure were fixed to 300 K and 1 atm, respectively. A time-step of 1 fs was applied throughout the molecular dynamics simulations. In dependent simulation runs, three colloid models were explored in ethanolic solution, mimicked by a cubic cell of (~10 nm)^3^ comprising 8442 ethanol molecules and subjected to periodic boundary conditions.

The association of surfactant molecules to our nanocrystal models was treated in full analogy to the uptake of ions to a forming aggregate as reported earlier [8,10,11]. Transferring the Kawska-Zahn approach to surface functionalization we thus mimic surfactant diffusion to the nanoparticle implicitly by applying random incoming vectors and random surfactant molecule orientation. Placing surfactant molecules in 1.5 nm distance from the nanoparticle, we then explore molecular docking from detailed molecular simulation. This is first performed in absence of solvent molecules (keeping the nanoparticle fixed) to obtain a putative association structure from simple energy minimization. Only this first part of the association step is performed without solvent to boost solute migration to the nanoparticle. In the next step, the nanoparticle-surfactant system is immersed into ethanolic solution and propagated by simulated annealing runs to allow the overall system (including solvent and Na^+^ counter ions) to relax. The sodium ions are initially placed randomly. Subsequently, their positions are propagated by performing nanosecond-scale molecular dynamics simulations to ensure convergence of the ionic distribution in the simulation cell. Three independent runs of this kind were performed to provide at least qualitative account for the abundance of possible nanoparticle-surfactant structures possible even for a single ZnO/Zn(OH)_2_ nanorod model.

## Results

**Fig 1:**
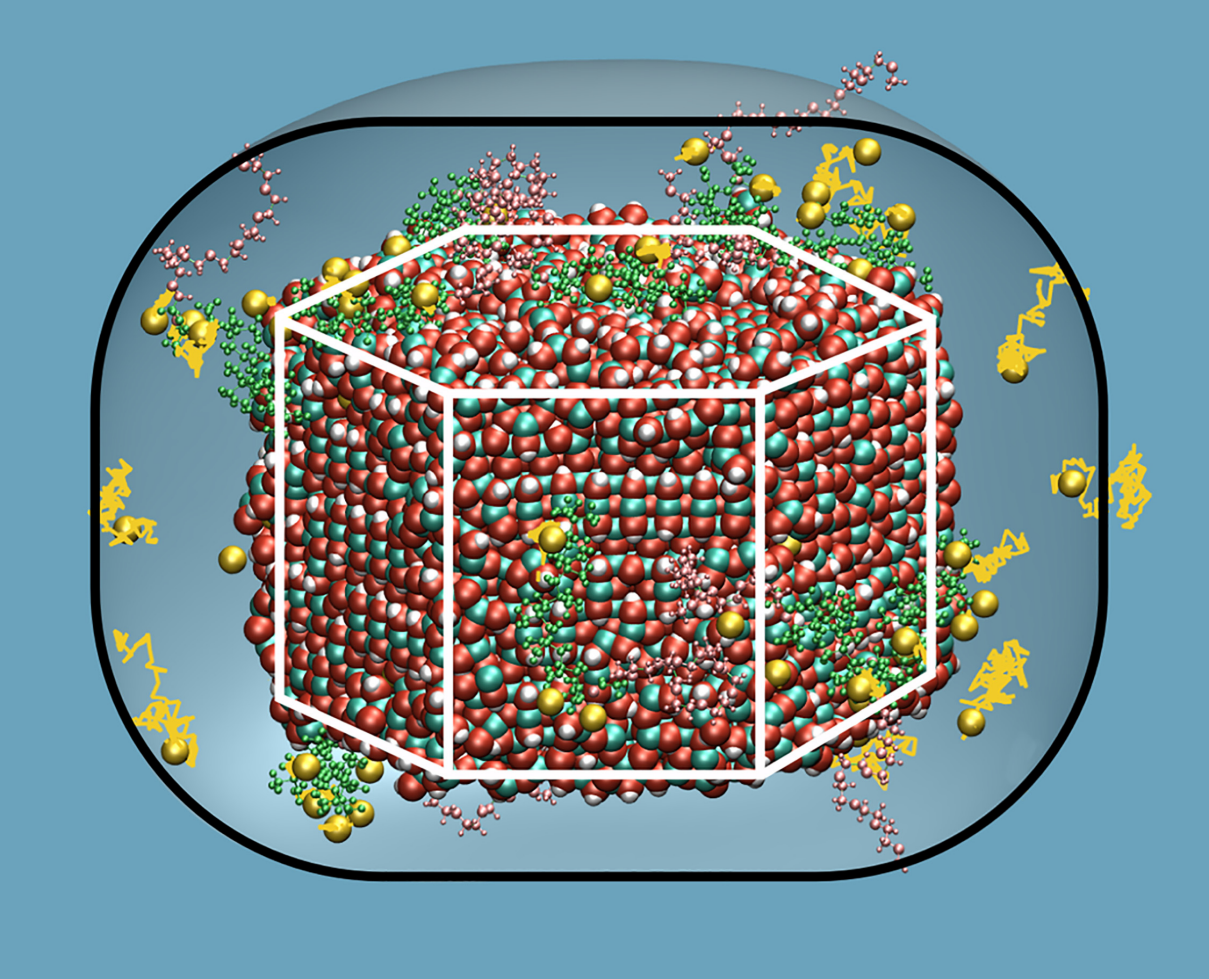
Illustration of the colloid model II highlighting the pathways of Na^+^ counterions (yellow) within 0.5 ns. While the solvent is not shown for clarity, the effective colloid dimensions including the halo is illustrated as a surrounding surface. Atom colors: Zn (cyan), O (red), H(white), Na(yellow). Polymer colors: EO (pink), MA (green).

The resulting colloid models differ in terms of surfactant association sites and the overall number of (EO_8_-b-MAA_8_)^8-^ polymers that stably bind to the ZnO/Zn(OH)_2_ nanoparticle. Surfactant association is dominated by the acrylate units which form a COO^-^ Zn^2+^ salt bridge and up to 1–2 COO^-^ OH^-^ hydrogen bonds per methylacrylate monomer. On the other hand, the PEO chains were found to not bind to the ZnO surface, but fluctuate considerably and are best described as dangling into the embedding solution (Fig 1). While association to the faces normal to the polar axis of the ZnO crystallite is only slightly preferred to binding laterally, we however observe a strong favoring of the top, i.e. (0001) over the bottom face indicative of selective binding of the negatively charged surfactants to (partially) compensate the dipole moment of the ZnO nanoparticle [12]. Statistics for binding to top, bottom and lateral faces are given for all model systems in Table 1.

**Table 1:** Ratio of surface areas normal to the polar axis of ZnO with respect to lateral faces and absolute numbers of surfactants bound to the top, bottom and lateral faces.

Model	Ratio of surface areas $\frac{\left\{0001\right\}}{\left\{10\bar{1}0\right\}}$	EO_8_-b-MAA_8_bound to: (0001)	EO_8_-b-MAA_8_bound to: $\left(000\bar{1}\right)$	EO_8_-b-MAA_8_bound to: $\left\{10\bar{1}0\right\}$	*Normalized ratio* $\frac{n_{\left\{0001\right\}}}{0.705\cdot n_{\left\{10\bar{1}0\right\}}}$
**I**	0.705	3	0	7	1.2
**II**	0.705	5	1	4	4.3
**III**	0.705	4	2	9	1.9

For comparison of association to the top and to lateral faces, the numbers of surfactants were put in relation to the corresponding surface areas.

**Fig 2:**
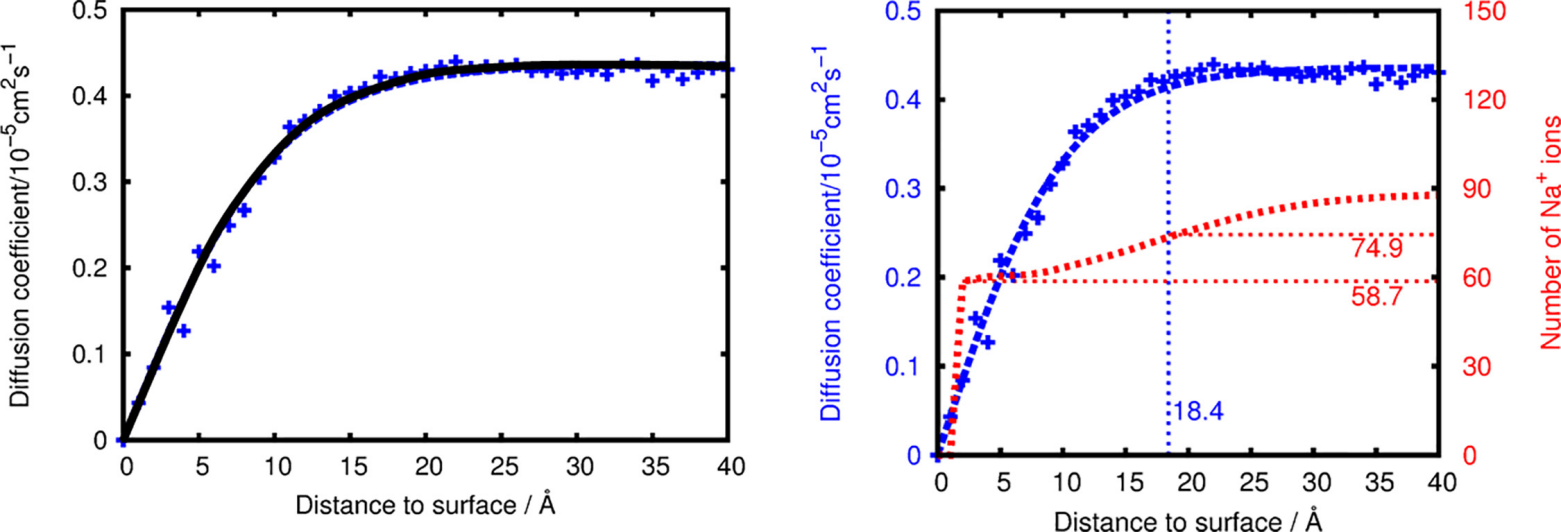
Left: Diffusion coefficient (blue) of the Na^+^ ions as a function of the distance to the colloid and fitted switching function (black) used for quantification of the degree of ion association to the colloid (model II). Right: same as left, but additionally illustrating the number of Na^+^ ions (red curve). The vertical blue line denotes the surface distance delimiter discriminating halo and bulk counter ions. The horizontal red lines point out the number of counter ions directly associated with the particle (58.7), and the total number of Na^+^ ions including the halo (74.9). All data was averaged over 100 ns. See S1 Fig for analogous plots of models I and III.

The counter ions (Na^+^) were placed in the solution and allowed to diffuse freely. While ion distribution was found to reach a dynamic equilibrium after a few tens of nanoseconds, additional 100 ns runs were used for the analyses described in the following. While the number of sodium ions was chosen to fully compensate the colloid charges, only a fraction form stable COO^-^ Na^+^ salt bridges and thus ‘behave’ akin to the surfactant molecules. The remaining sodium ions were found to be loosely associated and are best described as a dynamic equilibrium of Na^+^ association to surfactant molecules, migration within a nm-sized halo around the nanoparticle and dispersion into the bulk solution (Fig 1). To quantify this phenomenon we calculated the Na^+^ diffusion coefficient as a function of the ion-colloid distance. Fig 2A shows the continuous increase of ionic mobility within a distance of ~1.8 nm and a plateau region at farer distance from the colloid. While the latter corresponds to sodium ions not associated to the nanoparticle, Na^+^ that bind to the nanoparticle or its surfactants molecules exhibit a diffusion constant of zero and may thus be considered as surfactants (hence reducing the net charge of the colloid drastically). In-between, that is from 0 to 1.8 nm distance, Na^+^ ions are loosely associated and constitute a positively charged halo which also reduces the effective charge of the colloid.

We suggest to use the distance dependence of ionic mobility for quantification of the degree of Na^+^ association to the nanoparticle. For this purpose, we fitted the diffusion constants to a switching function f_switch_(r):

$$D\left(r\right)=D_{bulk\;solution}\cdot f_{switch}\left(r\right)=D_{bulk\;solution}\cdot \text{tanh}\left(a\cdot r\right)$$ (1)

where the use of a tangents hyperbolicus as the functional form of the switching function allows accurate fitting using a single parameter *a*. The same switching function is applied to attribute a weighted charge to the effective charge of the colloid:

$$q_{halo}^{Na+,effective}=+1\cdot \left\{1-f_{switch}\left(r\right)\right\}\;;\;Q_{colloid}^{effective}=Q_{surfac\;\text{tan}\;ts}+\sum_{i}q_{halo}^{Na+,effective}\left(r_{i}\right)$$ (2)

This allows a reasonable estimate of the effectively colloid charge including contributions from its halo of loosely associated counter ions (Fig 2B). Moreover, we suggest the fitted switching functions for assessing the effective radius of the colloids in solution. As the switching function reaches 1 only for infinite distance, we propose 0.95 as delimiter for discriminating the halo from the bulk solution. This value is somewhat arbitrary, and error margins of ± 0.1nm result from considering alternative switching function delimiters of 0.9 or 0.975, respectively. For all three models investigated, the effective radii and net charges are reported in Table 2.

**Table 2:** Number of block-copolymers and sodium ions bound as surfactants to the nanoparticles, number of charge carriers and thickness of the halo of counterions, effective radius and colloid charge, net dipole moment and polarizability as assessed for all model systems investigated.

Model	EO_8_-b-MAA_8_bound	Na^+^ bound	<Na^+^>in halo	*d*(halo) / Å	*R* ^*eff*^	$Q_{colloid}^{effective}$ / e	〈*p*〉/ e Å	α / eÅ^2^ V^-1^
**I**	10	50	17.5	19.7	50.5	-25.9	240.1	3.67 10^5^
**II**	11	59	16.2	18.4	49.1	-25.7	233.6	4.70 10^5^
**III**	15	90	15.0	17.8	48.4	-14.0	275.8	5.09 10^5^

**Fig 3:**
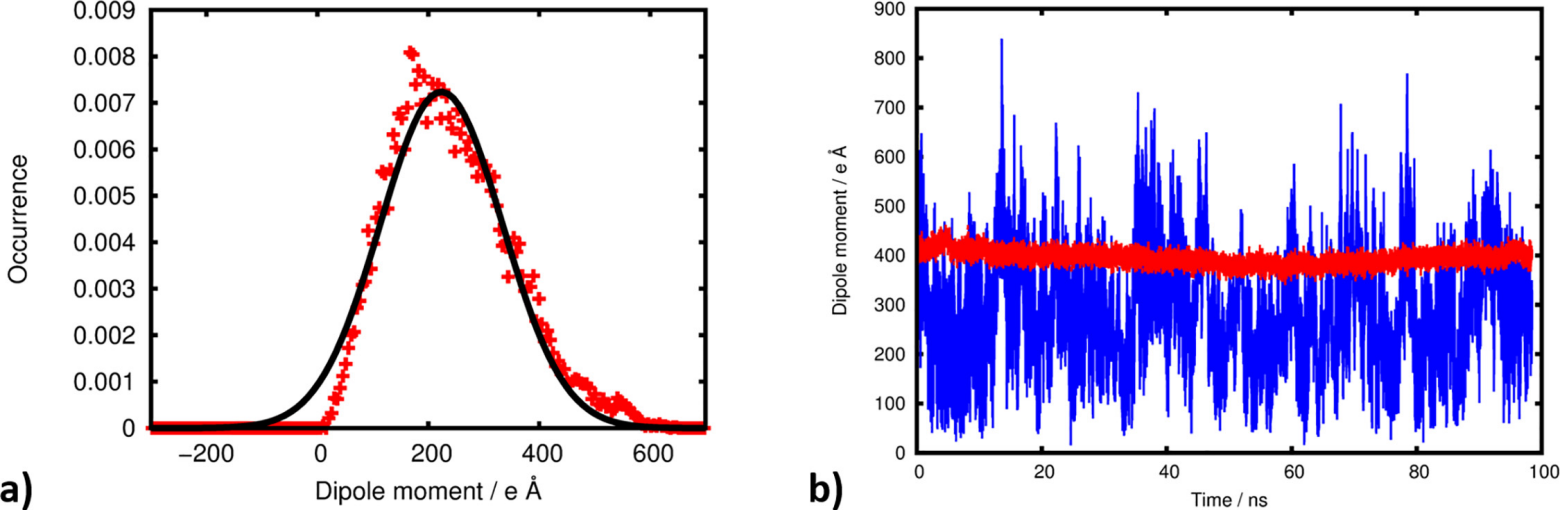
Occurrence profile a) of the dipole moment as sampled from the solvated colloid model II. The width of the Gaussian fit (black curve) is used to estimate the polarizability of the halo of counterions. See S2 Fig for analogous plots of models I and III. The dipole moment b) of the ZnO/Zn(OH)_2_ core-shell nanoparticle (red curve) and the colloid including surfactants and the halo of counterions (blue curve) are shown as functions of time. In agreement with ab initio calculations related to bulk ZnO crystal we observe that surface passivation with hydroxides already reduces the dipole moment by a factor of almost 10 [10]. Surfactants and counterions lead to further reduction by about 50%.

The above considerations refer to the predominant colloid-colloid interactions, i.e. Coulomb repulsion because of the negative net charges. However, the mobility of the sodium ions within the ~1.8 nm sized halo around the nanoparticle also allows for a molecular scale account of the van-der-Waals interactions. Using the effective charge model denoted in Eq (2), we calculated the dipole moments of the colloids in solution as functions of time. The corresponding occurrence profiles show i) a net dipole moment stemming from ZnO, and only partially compensated by hydroxylation and inhomogeneous distributions of the surfactants and counterions, and ii) considerable fluctuations of the overall dipole moment which is attributed to fluctuations within the halo of Na^+^ countercharges (Fig 3). Finding ii) reflects the variability of the dipole moment Δp = p(t)—<p> that arises from the distribution of mobile counterions in the solvation halo and thus reflects a general phenomenon of any charged colloid systems. Without actually applying an external electric field, our simulations allow the calculation of colloid polarizability α by relating the energy of the dipole field Δp stemming from polarizing the halo of counterions to Boltzmann statistics employed for the occurrence profile h(Δp) as illustrated in Fig 3. This leads to:

$$h\left(\Delta p\right)=h_{0}\cdot e^{-\frac{{\left(p-\langle p\rangle \right)}^{2}}{2\sigma }}=h_{0}\cdot e^{-\frac{\frac{1}{2}\alpha^{-1}{\left(\Delta p\right)}^{2}}{k_{B}T}}$$ (3)

For all of the three colloid solutions investigated, we find a nice Gaussian type occurrence profile of the dipole moment and were thus able to assess the polarizability α at good accuracy (Fig 3A). Strikingly, colloid polarizability is constant within very small margins, whilst the values of the effective charge and the dipole moments differ considerably within our set of models (Table 2). This may be rationalized by the roughly equal thickness and charge density of the halo of counterions, each comprising 15–18 Na^+^ ions. As a simple estimate for transferring our findings to different sizes of ZnO nanoparticles, it is thus intuitive to assume that the halo thickness remains constant for different nanoparticle sizes, while the number of *mobile* ions in the halo and thus polarizability should scale linearly with the particle surface area.

## Conclusion

In conclusion we elucidated the atomic mechanisms of ZnO nanoparticle stabilization by charged surfactants and its interplay with Na^+^ counterions from the embedding ethanolic solution. A large fraction of the sodium ions tends to bind to the nanoparticle-surfactant molecules system, and may hence be considered as surfactants as well. However, within a 1.8 nm sized halo around the colloid, we identified loosely associated Na^+^ which account for a polarizable ‘cloud’ of charges. Assessment of the underlying polarizability from molecular simulation—and thus molecular scale insights into van-der-Waals interactions of the colloids—is outlined from inexpensive sampling of the spontaneous fluctuations of the dipole moment.

While our simulations were based on a limited set of colloid models and one type of solvent (ethanol) only, from a qualitative viewpoint it is nevertheless intuitive to suggest the observed mechanisms to be of general relevance for rationalizing electrostatic nanoparticle shielding. We would be enthusiastic to offer our ZnO-polymer data for comparison with experiments—or to provide similar modelling work for other systems.

## Supporting Information

S1 Fig Diffusion coefficient (blue) of the Na^+^ ions as a function of the distance to the colloid and number of Na^+^ ions (red curve) for models I-III. All data was averaged over 100 ns.
(TIF)

S2 Fig Occurrence profile of the dipole moments as sampled from the solvated colloid models I-III. The width of the Gaussian fits is used to estimate the polarizability of the halo of counterions.
(TIF)

S3 Fig EO_8_-*b*-MAA_8_, hydrogen atoms are omitted to increase readability. Numbering of oxygen and hydrogen in S1 Table w.r.t. neighboring carbon atom.
(TIF)

S1 Table Force-field parameters used for Zinc-O (oxide/hydroxide) interactions. (DOCX)

S2 Table RESP charges and corresponding GAFF types of the EO_8_-*b*-MAA_8_ model. (DOCX)

S1 Text Preparation of the model system, interaction models and additional data analyses. (DOC)
