# Supplementary figures and images for: Molecular Mechanisms of ZnO Nanoparticle Dispersion in Solution: Modeling of Surfactant Association, Electrostatic Shielding and Counter Ion Dynamics
Source: PLoS One. 2015 May 11;10(5):e0125872. doi: 10.1371/journal.pone.0125872 (PMC4427181; doi:10.1371/journal.pone.0125872)

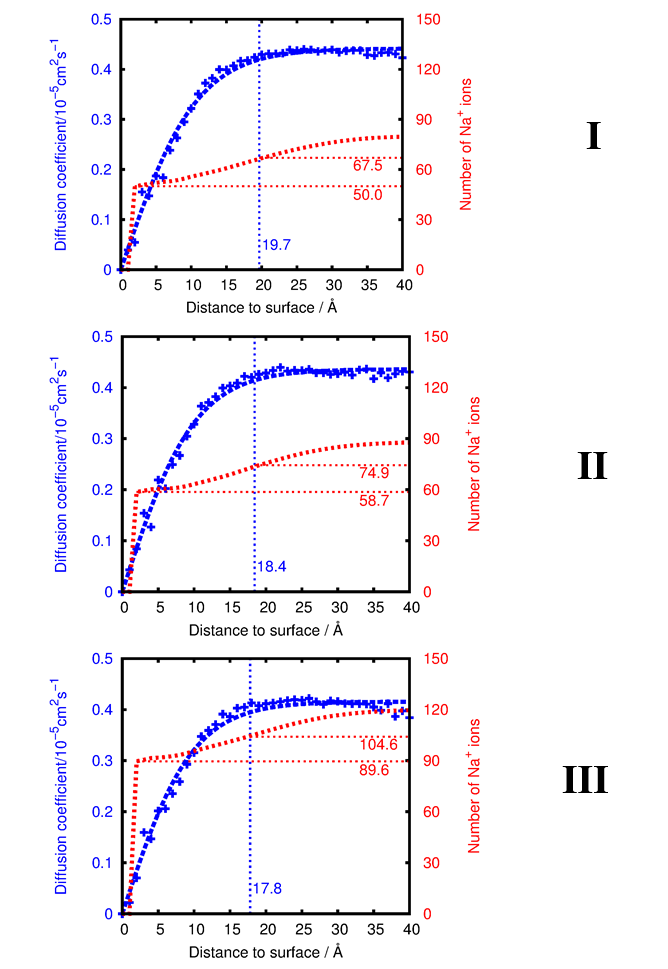

Supplement: S1 Fig — All data was averaged over 100 ns. (TIF) [file pone.0125872.s001.tif]

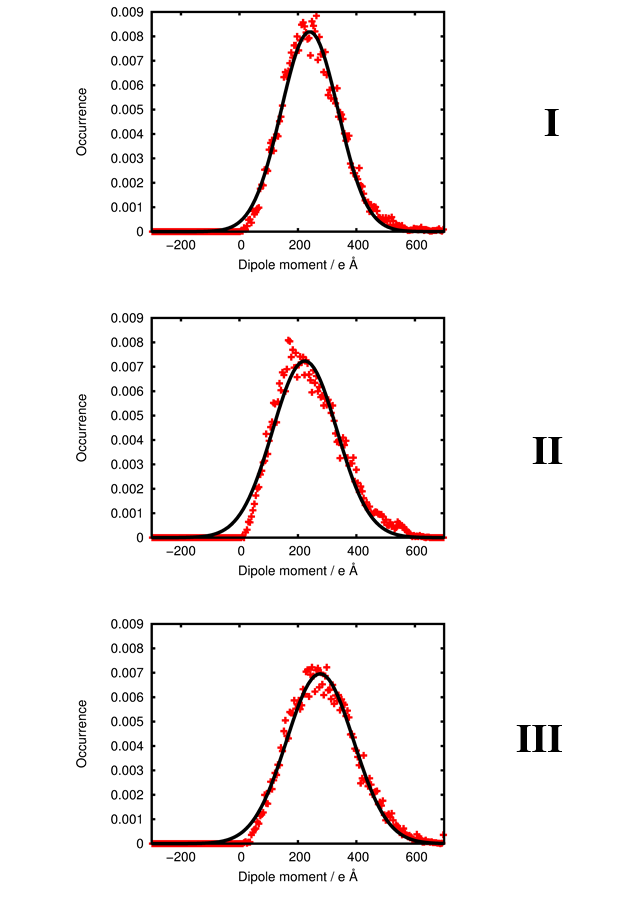

Supplement: S2 Fig — The width of the Gaussian fits is used to estimate the polarizability of the halo of counterions. (TIF) [file pone.0125872.s002.tif]

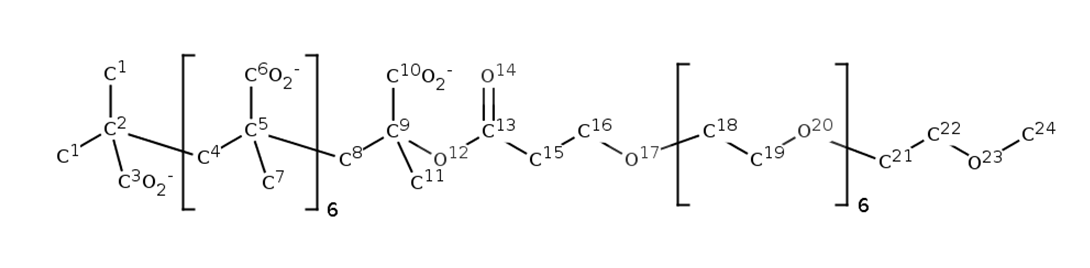

Supplement: S3 Fig — Numbering of oxygen and hydrogen in S1 Table w.r.t. neighboring carbon atom. (TIF) [file pone.0125872.s003.tif]
