# Supplementary material for: Molecular Mechanisms of ZnO Nanoparticle Dispersion in Solution: Modeling of Surfactant Association, Electrostatic Shielding and Counter Ion Dynamics
Source: PLoS One. 2015 May 11;10(5):e0125872. doi: 10.1371/journal.pone.0125872 (PMC4427181; doi:10.1371/journal.pone.0125872)
Supplement: S1 Table — (DOCX) [file pone.0125872.s004.docx]

|  | **A / kJ mol^-1^** | **ρ / Å** | **C / Å^6^ kJ mol^-1^** |
| --- | --- | --- | --- |
| **Zn − Zn** | 0.0 | 0.0 | 0.0 |
| **O − O** | 2,196,420.11 | 0.149 | 10,825.65 |
| **Zn − O** | 67,568.65 | 0.3372 | 0.0 |
