# Supplementary material for: Molecular Mechanisms of ZnO Nanoparticle Dispersion in Solution: Modeling of Surfactant Association, Electrostatic Shielding and Counter Ion Dynamics
Source: PLoS One. 2015 May 11;10(5):e0125872. doi: 10.1371/journal.pone.0125872 (PMC4427181; doi:10.1371/journal.pone.0125872)
Supplement: S2 Table — (DOCX) [file pone.0125872.s005.docx]

|  | **q / e** | **GAFF type** |
| --- | --- | --- |
| **C^1^** | -0.5058 | C3 |
| **H^1^** | 0.0230 | HC |
| **C^2^** | 1.3243 | C3 |
| **C^3^** | -0.8898 | C2 |
| **O^3^** | -0.2308 | O |
| **C^4^** | 0.0201 | C3 |
| **H^4^** | 0.0038 | HC |
| **C^5^** | 0.1629 | C3 |
| **C^6^** | -0.0938 | C2 |
| **O^6^** | -0.4059 | O |
| **C^7^** | -0.2655 | C3 |
| **H^7^** | -0.0065 | HC |
| **C^8^** | 0.9350 | C3 |
| **H^8^** | -0.4971 | HC |
| **C^9^** | 0.9342 | C3 |
| **C^10^** | -1.1810 | C2 |
| **O^10^** | -0.0266 | O |
| **C^11^** | 0.3928 | C3 |
| **H^11^** | -0.1849 | HC |
| **O^12^** | -0.4969 | OS |
| **C^13^** | 0.7882 | C |
| **O^14^** | -0.4887 | O |
| **C^15^** | 0.8458 | C3 |
| **H^15^** | 0.1702 | HC |
| **C^16^** | 0.7000 | C3 |
| **H^16^** | -0.1229 | H1 |
| **O^17^** | -0.4277 | OS |
| **C^18^** | 0.4502 | C3 |
| **H^18^** | -0.1402 | H1 |
| **C^19^** | 0.2552 | C3 |
| **H^19^** | -0.0724 | H1 |
| **O^20^** | -0.2800 | OS |
| **C^21^** | 0.1452 | C3 |
| **H^21^** | 0.0008 | H1 |
| **C^22^** | 0.0738 | C3 |
| **H^22^** | 0.0413 | H1 |
| **O^23^** | -0.3591 | OS |
| **C^24^** | 0.1752 | C3 |
| **H^24^** | -0.0070 | H1 |
