# Supplementary material for: Molecular Mechanisms of ZnO Nanoparticle Dispersion in Solution: Modeling of Surfactant Association, Electrostatic Shielding and Counter Ion Dynamics
Source: PLoS One. 2015 May 11;10(5):e0125872. doi: 10.1371/journal.pone.0125872 (PMC4427181; doi:10.1371/journal.pone.0125872)
Supplement: S1 Text — (DOC) [file pone.0125872.s006.doc]

Supporting Information

**Preparation of ZnO/Zn(OH)_2_ models from up-scaling**

The inner core of the nanoparticle was chosen as a hexagonal prism (of about 5 nm diameters along the a,b and c axes, respectively) cut from the ZnO crystal structure. To mimic a realistic surface, O^2-^ ion protonation (or more precisely incomplete OH^-^ deprotonation) and charge compensation by Zn^2+^ deficiencies were carefully considered. This was based on our earlier study of ZnO nucleation from solution [S1], suggesting the absence of exposed O^2-^ ions at the surface leading to a single Zn(OH)_2_-type layer as the outer shell of Zn_x_O_y_(OH)_z_ aggregates as a general building rule. The prepared nanorod model comprises 5422 Zn^2+^, 4860 O^2-^ and 1124 OH^-^ ions and exhibits no net charge.

**Analyses of diffusion constants for all three model systems investigated**

Fig. A

Diffusion coefficient (blue) of the Na^+^ ions as a function of the distance to the colloid and number of Na^+^ ions (red curve) for models I-III. All data was averaged over 100 ns.

**Analyses of colloid dipole moments and polarizability for all three model systems investigated**

Fig. B

Occurrence profile of the dipole moments as sampled from the solvated colloid models I-III. The width of the Gaussian fits is used to estimate the polarizability of the halo of counterions.

**Simulation parameters**

We used the DLPOLY code [S2] to carry out the molecular dynamics simulations. The Berendsen thermostat and barostat were employed to impose room temperature and ambient pressure, with relaxation times of 0.5 ps and 5.0 ps, respectively. Due to the large size of the model system, extended summation of forces in real space is preferred over Ewald summation. We thus used a shifted coulombic potential with a generous cutoff of 15Å.

Different sets of empirically derived all-atom force field parameters were combined.

For the Zn^2+^ − O^2-^ interactions we employed the Buckingham-type parameter set developed by Lewis and Catlow [S3]:

For the EO_8_-*b*-MAA_8_ model we used the GAFF parameter set [S4], determining the partial charges with the restrained electrostatic potential (RESP) methodology [S5], with dedicated modeling for the terminal EO- and MAA-terminal-monomers. Ethanol parameters were taken from the OPLS-AA set [S6].

Fig. C

EO_8_-*b*-MAA_8_, hydrogen atoms are omitted to increase readability. Numbering of oxygen and hydrogen in table S2 w.r.t. neighboring carbon atom.

Table A

Force-field parameters used for Zinc-O (oxide/hydroxide) interactions.

Table B

RESP charges and corresponding GAFF types of the EO_8_-*b*-MAA_8_ model.

[Sl] A.Kawska, P.Duchstein, O.Hochrein, D.Zahn, “Atomistic Mechanism of ZnO Nucleation from Ethanolic Solution: Ion Association, Proton Transfer and Selforganization”, Nanoletters, 8 (2008) 2336-2340.

[S2] W.Smith, I.T.Todorov, M.Leslie, “The DL_POLY molecular dynamics package”, Zeitschrift für Kristallographie 220 (2005), 563-566

[S3] G.V.Lewis, C.R.A. Catlow, “Potential models for ionic oxides”, Journal of Physics C: Solid State Physics, 18 (1985), 1149-1161.

[S4] J.Wang, R.M.Wolf, J.W.Caldwell, P.A.Kollman, D.A.Case, “Development and testing of a general amber force field”, Journal of computational chemistry, 25 (2004), 1157-1174

[S5] C.I.Bayly, P.Cieplak, W.Cornell, P.A.Kollman, “A well-behaved electrostatic potential based method using charge restraints for deriving atomic charges: the RESP model”, Journal of Physical Chemistry, 97 (1993), 10269-10280

[S6] W.L.Jorgensen, D.S.Maxwell, J.Tirado-Rives, “Development and Testing of the OPLS All-Atom Force Field on Conformational Energetics and Properties of Organic Liquids”, Journal of the American Chemical Society, 118 (1996), 11225-11236
